# Microstructural brain abnormalities in HIV+ individuals with or without chronic marijuana use

**DOI:** 10.1186/s12974-020-01910-5

**Published:** 2020-08-06

**Authors:** Hannah A. Wang, Hua-Jun Liang, Thomas M. Ernst, Kenichi Oishi, Linda Chang

**Affiliations:** 1 Department of Diagnostic Radiology and Nuclear Medicine, University of Maryland School of Medicine, 670 W. Baltimore Street, HSF III, Baltimore, MD 21201 USA; 2 Department of Neurology, Johns Hopkins University School of Medicine, Baltimore, MD USA; 3 Department of Medicine, University of Hawaii, John A. Burns School of Medicine, Honolulu, HI USA; 4 Department of Radiology, Johns Hopkins University School of Medicine, Baltimore, MD USA; 5 Department of Neurology, University of Maryland School of Medicine, Baltimore, MD USA

**Keywords:** Fractional anisotropy, Diffusivity, HIV, Marijuana, DTI, Cognition

## Abstract

**Objective:**

Cognitive deficits and microstructural brain abnormalities are well documented in HIV-positive individuals (HIV+). This study evaluated whether chronic marijuana (MJ) use contributes to additional cognitive deficits or brain microstructural abnormalities that may reflect neuroinflammation or neuronal injury in HIV+.

**Method:**

Using a 2 × 2 design, 44 HIV+ participants [23 minimal/no MJ users (HIV+), 21 chronic active MJ users (HIV + MJ)] were compared to 46 seronegative participants [24 minimal/no MJ users (SN) and 22 chronic MJ users (SN + MJ)] on neuropsychological performance (7 cognitive domains) and diffusion tensor imaging metrics, using an automated atlas to assess fractional anisotropy (FA), axial (AD), radial (RD), and mean (MD) diffusivities, in 18 cortical and 4 subcortical brain regions.

**Results:**

Compared to SN and regardless of MJ use, the HIV+ group had lower FA and higher diffusivities in multiple white matter and subcortical structures (*p* < 0.001–0.050), as well as poorer cognition in Fluency (*p* = 0.039), Attention/Working Memory (*p* = 0.009), Learning (*p* = 0.014), and Memory (*p* = 0.028). Regardless of HIV serostatus, MJ users had lower AD in uncinate fasciculus (*p* = 0.024) but similar cognition as nonusers. HIV serostatus and MJ use showed an interactive effect on mean diffusivity in the right globus pallidus but not on cognitive function. Furthermore, lower FA in left anterior internal capsule predicted poorer Fluency across all participants and worse Attention/Working Memory in all except SN subjects, while higher diffusivities in several white matter tracts also predicted lower cognitive domain *Z*-scores. Lastly, MJ users with or without HIV infection showed greater than normal age-dependent FA declines in superior longitudinal fasciculus, external capsule, and globus pallidus.

**Conclusions:**

Our findings suggest that, except in the globus pallidus, chronic MJ use had no additional negative influence on brain microstructure or neurocognitive deficits in HIV+ individuals. However, lower AD in the uncinate fasciculus of MJ users suggests axonal loss in this white matter tract that connects to cannabinoid receptor rich brain regions that are involved in verbal memory and emotion. Furthermore, the greater than normal age-dependent FA declines in the white matter tracts and globus pallidus in MJ users suggest that older chronic MJ users may eventually have lesser neuronal integrity in these brain regions.

## Introduction

HIV infection is associated with chronic neuroinflammation, which contributes to cognitive dysfunction [1] and various brain structural and functional abnormalities in people living with HIV (PLWH) [2]. Cannabis, or marijuana (MJ), is the most commonly abused illicit drug worldwide and in the USA [3] and is used much more often in PLWH than in the general population (26.4% vs.16%) [4, 5]. With the legalization of cannabis for recreational MJ use in many states in the USA and in other countries, the prevalence of MJ use among PLWH has continued to increase [4].

Despite the highly prevalent MJ use by PLWH, data regarding whether HIV infection and MJ use may lead to additive or interactive effects on brain function or pathology remain scant and controversial [6]. Several studies found no independent or additive effects on cognitive deficits with chronic MJ users with or without HIV infection [7–9]. However, MJ use in PLWH was also found to have worse motor learning deficits [10], as well as better verbal fluency and learning [11].

Similar inconsistent findings were reported in MJ users without HIV infection. Chronic MJ users showed poorer learning, memory, attention, executive planning, and lower intelligence quotient (IQ) [12–14]. However, deficits in learning and memory normalized within a month after abstinence from MJ use [12, 13]. Further, MJ users did not show greater cognitive decline compared with nonusers [15, 16], except for those with adolescent onset of MJ use [12, 14]. In addition, chronic MJ use may lead to apathy and lack of motivation [17]. Although chronic MJ use may suppress the immune system [18], MJ use in PLWH did not influence viral suppression by cART [19], adherence to cART [20], or mortality [21].

Few neuroimaging studies evaluated the combined effects of HIV infection and MJ use, but the findings were variable. For instance, chronic MJ use in PLWH did not show additional effects on brain atrophy [8] but had interactive effects on brain glutamate levels on proton MR spectroscopy [7]. HIV + MJ smokers also showed greater brain activation in frontal-insular regions compared to HIV+ individuals or MJ users [22]. Several diffusion tensor imaging (DTI) studies evaluated MJ users and found disrupted microstructural integrity in corpus callosum (CC), superior longitudinal fasciculus (SLF), thalamic radiation and uncinate fasciculus (UNC), as well as abnormal structural connectivity to the orbitofrontal cortex (OFC) [23–26]. Conversely, no group difference between recreational MJ users and nonusers on white matter integrity was also reported, except for an association between lesser white matter coherence in those with earlier age of first use [27]. Whether chronic MJ use influences brain microstructure in PLWH is unknown.

Therefore, the current study evaluated whether brain microstructure differs between HIV+ individuals with and without chronic active MJ use (≥ 3 times/week for past 2 years or longer). All HIV+ participants in the current study were maintained on combined antiretroviral therapy (cART) regimens. We hypothesize that: (1) Consistent with prior reports [7–9], HIV+ individuals but not MJ users would have poorer cognitive function compared to seronegative non-MJ users (SN), with no interactive or synergistic effects between HIV+ and MJ use on cognitive performance. (2) Based on aforementioned DTI studies, HIV+ subjects would show lower FA and higher diffusivities (AD, RD, and MD) compared to SN, while MJ users would show minimal or no abnormalities, on DTI in the major fiber tracts and subcortical gray matter. Hence, we expected HIV + MJ users to show minimal or no additional effects on DTI metrics in these brain regions compared to either HIV+ subjects without MJ use or SN-MJ users.

## Methods

### Participants

Using a 2 × 2 design, 90 participants (ages 18–70 years), including 24 SN participants with no MJ use, 22 SN with chronic MJ use (SN + MJ), 23 HIV+ participants with minimal or no MJ use (HIV+), and 21 HIV+ with chronic active MJ use (HIV + MJ), were included in this study. All participants were recruited from the local community, by referrals, on-line advertisements, or flyer postings and were screened initially by telephone. Three hundred thirty individuals were screened initially; 182 (55%) potentially eligible participants were invited for further in-person screening. Each signed a written consent form after being verbally informed of the study aims and requirements. The protocol and the consent form were approved by the Cooperative Institutional Review Board of the University of Hawaii and The Queen’s Medical Center and were Health Insurance Portability and Accountability Act (HIPAA) compliant.

Each participant was additionally screened with detailed medical and drug use histories, medical records reviews, and underwent physical and neuropsychiatric examinations by trained research staff and physicians to ensure they fulfilled the study criteria. 127/182 (70%) participants fulfilled all study criteria, but only 90/127 (71%) of those completed the study. They were men or women of any ethnicity, aged 18–70 years, and able to provide informed consent. SN participants were negative on the ClearView® COMPLETE HIV-1/2 test. HIV+ participants fulfilled these inclusion criteria: (1) HIV seropositive (with documentation from medical records) and (2) maintained on a stable combined antiretroviral therapy regimen for 6 months or longer (by self-report and verified by medical records whenever possible). MJ participants fulfilled these inclusion criteria: (1) chronic MJ use (> 3 times/week for > 2 years) and (2) negative urine toxicology screen for other drugs of abuse (methamphetamine, amphetamine, cocaine, benzodiazepine, barbiturates, and opiates, except for false positive tests from prescribed medications). Exclusion criteria for all participants were similar to those reported previously [7]: (1) history of co-morbid major psychiatric illness; (2) any confounding neurological disorder; (3) significantly abnormal laboratory tests (> 2 standard deviations); (4) moderate to severe substance use disorders (SUD) within the previous 2 years (Diagnostic Statistical Manual-5 SUD criteria, other than marijuana and/or tobacco use disorders); (5) positive urine toxicology screen on the day of visit, except for Δ9-tetrahydrocannabinol (Δ9-THC) in the MJ users; (6) pregnancy; (7) inability to read at the 8th grade level (on Wechsler Test of Adult Reading); and (8) contraindications for MRI studies.

### Image acquisition and processing

All participants were scanned on a 3 Tesla Siemens TIM Trio scanner (Siemens Medical Solutions, Erlangen, Germany). After a localizer, a sagittal 3-D magnetization-prepared rapid gradient-echo scan (TR/TE/TI = 2200/4.47/1000 ms; 1 mm isotropic resolution) and an axial fluid attenuated inversion recovery scan (FLAIR, TR/TE = 9100/84 ms, 3-mm slice thickness, 44 slices) were performed. All structural MR scans were reviewed by an experienced Neurologist (L.C.) to evaluate for any confounding gross structural abnormalities. Five of the 90 total participants had minor structural abnormalities: One had a small area of encephalomalacia in the right posterior parietal region; the second had several small gliotic lesions from old toxoplasma lesions in the anterior frontal lobes, the anterior cingulate cortex and thalamus; the third had a small old lacunar infarct in the ponto-cerebellar junction; the fourth had some small areas of white matter hyperintensities in the U-fibers in the parieto-occipital region; and the fifth had a small area of hyperintense signal at the right frontal and temporal lobe juncture along the Sylvian fissure. These abnormalities did not significantly impact the selected regions of interest (ROIs), based on comparisons of our findings with or without these 5 participants’ data. DTI scans were performed with *b* = 0 and 12 directions at 1000 s/mm^2^, TR/TE = 3700/88 ms, resolution 1.7 × 1.7mm^2^, 4 mm axial slices with 1-mm gap, and 4 repetitions.

Following motion correction [28], the tensor field for each individual brain was calculated using DTIStudio (www.MriStudio.org) and automatically fit to JHU-MNI atlas space using Large Deformation Diffeomorphic Metric Mapping [28, 29]. Fractional anisotropy (FA), and axial (AD, first eigenvalue), and radial diffusivity (RD, mean of second and third eigenvalues) were measured in anatomical regions defined in the JHU-MNI atlas [30]. Based on prior DTI studies that demonstrated regional white matter abnormalities in MJ users [23–26] and in HIV-infected individuals [2], FA, AD, and RD were assessed in the following 11 major white matter structures (18 including the subsections of each structure): corona radiata (anterior, superior, and posterior; or ACR, SCR, and PCR), corpus callosum (genu, body, and splenium; or GCC, BCC, and SCC), sagittal stratum (SS), SLF, superior fronto-occipital fasciculus (SFO), inferior fronto-occipital fasciculus (IFO), internal capsule (anterior and posterior limbs and retrolenticular part; or ALIC, PLIC, and RLIC), external capsule (EC), posterior thalamic radiation (PTR), UNC, and cingulum (connecting to the cingulate gyrus and to the hippocampus; or CGC and CGH). Due to excessively high proportion of crossing fibers, only FA and mean diffusivity (MD, average of the three eigenvalues) were assessed in the four subcortical regions [caudate, putamen, globus pallidus (GP), and thalamus].

### Neuropsychological testing

Cognitive function was assessed in 7 domains: (1) Learning was assessed with the Rey Auditory Verbal Learning Test (RAVLT, immediate recall) and the Rey-Osterreith Complex Figure Test (RCFT, immediate recall). (2) Memory was assessed with the RAVLT (delayed recall) and RCFT (delayed recall). (3) Executive function was assessed with the Delis-Kaplan Executive Function System (D-KEFS) Stroop Color-Word Interference Test and Trail-making (Number-Letter Switching). (4) Attention/Working Memory was assessed with the Wechsler Adult Intelligence Scale-Fourth Edition and Wechsler Memory Scale-Fourth Edition. (5) Speed of Information Processing was assessed with the D-KEFS Trail-making (Number Sequencing) Test, D-KEFS Stroop Color Naming Test, the mean simple reaction time from the California Computerized Assessment Package. (6) Design and Verbal Fluency were assessed with D-KEFS Design Fluency Test and D-KEFS Controlled Oral Word Association Test. (7) Fine motor skill was assessed with the Grooved Pegboard Test. The average duration to complete all the tests was 4 hours, with a break after the first half of the tests. *Z*-scores were generated for each domain, adjusted for age and education, based on a normative database from 547 SN healthy participants who were administered the same tests in a standardized manner in the same laboratory.

### HIV-associated neurocognitive disorder (HAND) or HAND-equivalent status

Following the guidelines from the Frascati criteria [31], we use the *Z*-scores generated for the 7 cognitive domains above, along with our clinical assessments, to evaluate all HIV+ individuals to determine whether they had HAND, and all SN controls whether they had HAND-equivalent cognitive status. Each HAND or HAND-equivalent participant was further subcategorized into asymptomatic neurocognitive impairment (ANI), mild neurocognitive disorder (MND), or HIV-associated dementia (HAD) or HAD-equivalent, based on the Frascati criteria that considered whether the cognitive impairment affected the subject’s self-reported mental acuity and daily functioning at work or at home [31]. The impact on the subject’s daily functioning was determined from the clinical assessment performed by a physician that included a detailed neuropsychiatric evaluation, along with the HIV dementia scale [32] and the Functional Activities Questionnaire [33].

### Statistics

All analyses were performed using R (version 3.5.2 https://www.R-project.org/). One-way analysis of variance (ANOVA), Chi-square, Mann-Whitney Test, Kruskal-Wallis Test, and Fisher’s Exact Test were used to compare the demographic measures and clinical variables depending on the variable types and distributions. Two-way analyses of co-variance (ANCOVAs) were performed to evaluate the independent and interactive effects of HIV serostatus and chronic active MJ use across the four groups, on the cognitive domain *Z*-scores and on DTI metrics in the 22 ROIs. Comparisons of the cognitive domain *Z*-scores were performed with 2-way ANOVA, without co-variates, since age and education level were already adjusted in the *Z*-scores. However, 2-way ANCOVA models for DTI metrics included covariates from variables that are known to or might have an influence on the DTI metrics, such as age, as well as the substance use variables that showed group differences across our subject groups, including percentage of lifetime tobacco users and percentage of regular (> once/week) alcohol users within the past month. A *p* value < 0.05 was considered significant for cognitive domain *Z*-scores. ROI-based analyses on DTI were also adjusted for multiple comparisons using the Benjamini-Hochberg procedure.

Exploratory correlations were performed between DTI metrics and cognitive domain *Z*-scores that showed group differences, using the following general linear models: cognitive domain *Z*-scores as dependent variables; DTI metrics, HIV-status, MJ use-status, and their 2-way and 3-way interactions as independent variables; and age as a covariate. Similar methods were used to explore the correlations between DTI metrics and age, HIV-related clinical variables, or MJ use patterns.

## Results

### Participant characteristics (Table 1)

All four groups had similar age, sex and racial distributions, socioeconomic status, years of education, and predicted verbal intelligence quotient (IQ). SN had the lowest depressive symptom scores on the CES-D across the four groups (*p* = 0.015). The two HIV+ groups had similar duration of HIV infection, current plasma RNA levels, nadir and recent CD4 cell counts, percentage of participants on stable combination antiretroviral therapy (cART) regimens, HIV dementia scores, and Karnofsky scales. The two MJ user groups also had similar age of first MJ use, duration of MJ use, daily MJ use, and total lifetime MJ usage. Although more MJ users reported lifetime tobacco use (*p* = 0.016) and regular recent alcohol use (> once/week within past month, *p* = 0.004) than non-MJ users, the four groups had similar total lifetime amount and duration of alcohol or tobacco use. Nevertheless, we included the percentage of lifetime tobacco use and percentage of regular recent alcohol use as covariates in the final model that evaluated the DTI metrics.

**Table 1:** Participant demographics and clinical characteristics (mean ± S.E.)

	SN nonusers (*N* = 24)	SN-MJ user (*N* = 22)	HIV nonusers (*N* = 23)	HIV + MJ user (*N* = 21)	*p* value
Age (years)	44.6 ± 2.8	45.3 ± 2.1	46.8 ± 2.4	46.3 ± 1.9	0.904^a^
Age range	(18.5–68.9)	(25.9–65.8)	(28.3–70.3)	(26–60.7)	
# Men (%)	21 (87.5%)	19 (86.4%)	22 (95.7%)	20 (95.2%)	0.567^b^
Education (years)	14.8 ± 0.5	13.7 ± 0.5	15.2 ± 0.4	14.2 ± 0.5	0.167^a^
WTAR Predicted Verbal IQ	109.8 ± 1.6	106.4 ± 2.1	107.9 ± 1.6	103.7 ± 1.9	0.103 ^a^
Race (W/As/B/NH/NA/Mixed)	12/4/1/1/1/5	13/0/1/1/0/3	11/7/1/0/0/5	13/3/1/1/0/4	0.629^b^
Index of Social Position (8–66)	36.3 ± 3.4	40.2 ± 3.8	35.8 ± 3.3	44.8 ± 3.6	0.233^a^
CES-Depression score (0–60)	5.9 ± 0.9	13.0 ± 3.3	14.3 ± 2.0	13.0 ± 1.9	**0.015**^**a**^
HIV disease-related					
Duration (months)	–	–	327.4 ± 29.9	339.4 ± 31.5	0.837^d^
# With Detectable HIV RNA (> 40 copies/mL, %)	–	–	3 (13.0%)	4 (19.1 %)	0.488^b^
Log plasma HIV RNA	–	–	1.8 ± 0.2	2.0 ± 0.3	0.420^c^
Plasma HIV RNA (copies/mL)^*^	–	–	4,064 ± 3,970	2,933 ± 1,774	0.814^c^
# (%) on combined antiretrovirals	–	–	23 (100%)	21 (100%)	
CD4 count (#/mm^3^)	–	–	511.9 ± 42.4	563.4 ± 65.4	0.514^d^
Nadir CD4 count (#/mm^3^)	–	–	250.6 ± 46.4	218.1 ± 46.0	0.622^d^
HIV dementia scale (0–16)	–	–	14.3 ± 0.5	14.2 ± 0.6	0.886^d^
Karnofsky score (0–100)	–	–	94.5 ± 1.4	92.9 ± 1.4	0.405^d^
# (%) with HAND or equivalent^**^	5 (20.8%)	6 (27.3%)	8 (34.8%)	6 (28.6%)	0.705^b^
Marijuana usage, median (range)					
Age at first use (years)	–	15.5 (8–39)	–	16 (9–40)	0.906^d^
Daily average use (g)	–	0.5 (0.02–3.5)	–	0.4 (0.04–3.6)	0.699^e^
Total lifetime use (kg)	–	3.5 (0.06–54.5)	–	3.2 (0.08–39.6)	0.941^c^
Duration of MJ use (years)	–	27.3 (1.9–45.3)	–	31.8 (3.7–41.7)	0.784^c^
Tobacco Smoking, median (range)					
# Lifetime *tobacco smokers* (%)	12 (50.0%)	18 (81.8%)	11 (47.8%)	17 (81.0%)	**0.016**^b^
# Smokers in the past month (%)	9 (37.5%)	13 (59.1%)	5 (21.7%)	8 (40.0%)	0.085^b^
Daily average use (g, range)	24.4 (0.5–76.3)	19.9 (0.002–61)	29.9 (0.01–61)	27.5 (2.0–39.3)	0.774^e^
Total lifetime use (pack-year)^¶^ (range)	10.8 (0.01–39.9)	16.1 (0.0001–59.3)	14.0 (0.0002–50.4)	16.4 (0.2–37.0)	0.733^e^
Duration of use (year, range)	14.8 (0.6–32.5)	23.9 (1–48.1)	17.9 (0.6–35.9)	25.8 (2.9–41.7)	0.241^e^
Duration of abstinence (months)^¶^	0 (0–390)	0 (0–483)	3 (0–332)	0 (0–370)	0.367^e^
Alcohol usage, median (range)					
# Lifetime alcohol users (%)	22 (91.7%)	19 (86.4%)	21 (95.5%)	20 (95.2%)	0.651^b^
Regular use (> 1/week) in the past month (%)	6 (25.0%)	14 (63.6%)	6 (27.3%)	7 (33.3%)	**0.004**^**b**^
Daily average use (mL)^¶^	9.4 (0.06–48.9)	12.3 (0.4–41.8)	6.1 (0.2–63.0)	7.7 (0.4–186.9)	0.632^e^
Total lifetime use (L, range)	71.5 (0.1–655)	110.3 (0.9–613)	86.8 (0.4–968)	56.6 (0.1–2491)	0.767^e^
Duration of use (years)^¶^	22.9 (2.3–46.5)	25.0 (5.7–45.5)	23.2 (9.0–46.6)	29.1 (0.9–44.8)	0.792^e^
Duration of abstinence (months)^¶^	0 (0–63)	0 (0–59)	1 (0–89)	0 (0–91)	0.197^e^

*p* values < 0.05 are bolded

*WTAR* Wechsler Test of Adult Reading, *CES-D* Center for Epidemiological Studies–Depression Scale, *Index of Social Position* assessed using the Hollingshead Four Factor Index of Social Position, *Race* White/Asian/Black/Native Hawaiian/Native American/more than one race

#Tobacco use status was missing in 5 participants; alcohol use status was missing in 3 participants

*Plasma HIV RNA was calculated from 3 (HIV+) and 4 (HIV + smoker) participants with detectable viruses

**HIV-associated neurocognitive disorder (HAND) diagnoses: HIV nonuser (3 ANI, 3 MND, 2 HAD), HIV + MJ users (2 ANI, 2 MND, 2 HAD), or HAND equivalent in the SN groups: SN nonuser (4 ANI and 1MND) and SN-MJ (4 ANI and 2 MND)

^¶^These variables were calculated only among the users

^a^ANOVA

^b^*χ*^2^

^c^Mann-Whitney *U*

^d^Student *t* test

^e^Kruskal-Wallis Test

### HIV and chronic MJ use on neuropsychological test performance (Fig. 1)

**Fig. 1:**
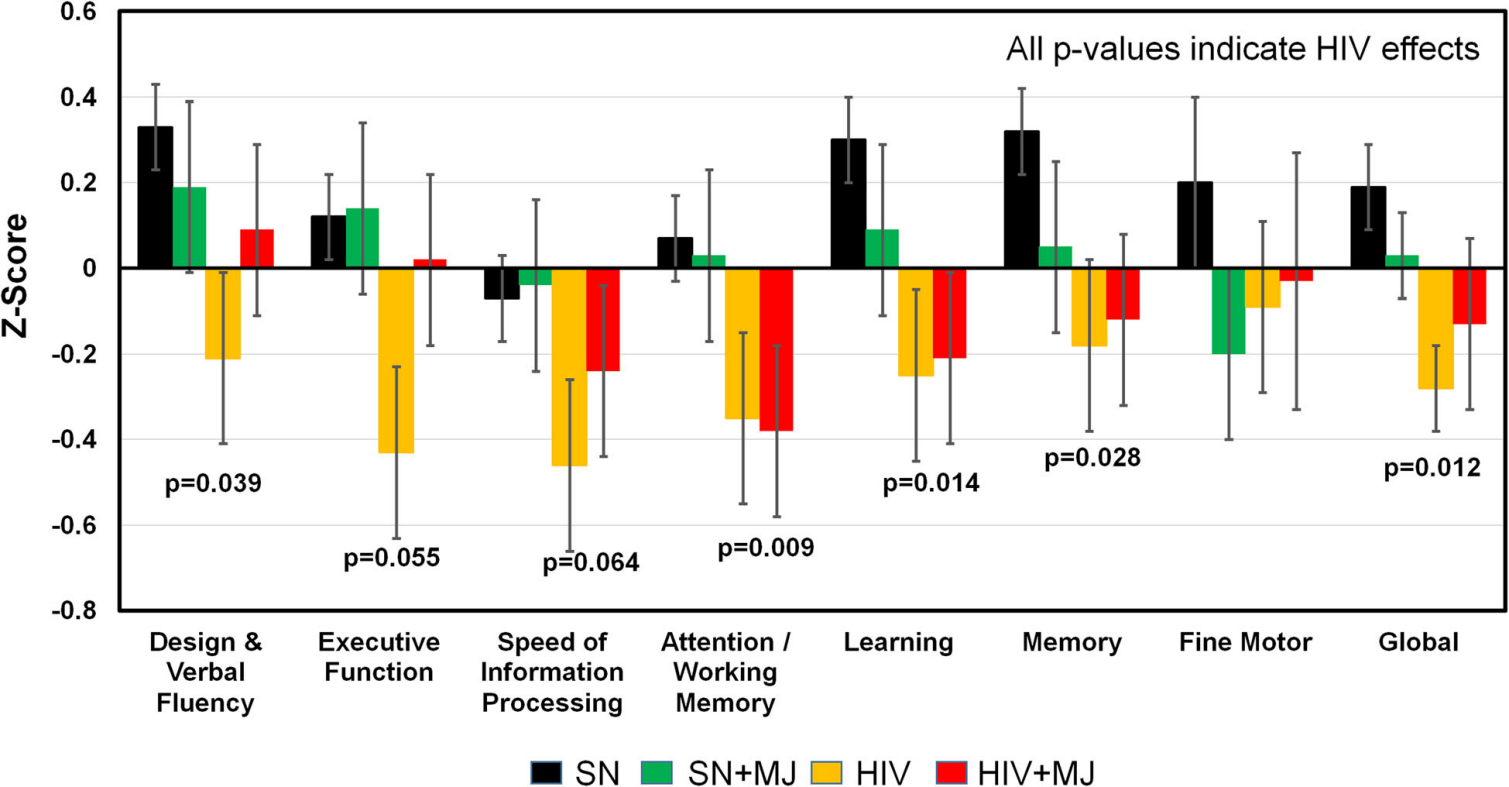
Cognitive domain *Z*-scores in the four study participant groups. Regardless of marijuana use, HIV+ participants had significantly poorer performance in the domains of Design and Verbal Fluency, Attention/Working Memory, Learning, and Memory, as well as the Global *Z*-score, compared to HIV-seronegative participants. Error bars = standard errors

Regardless of MJ use, HIV+ individuals had lower *Z*-scores than SN controls in the domains of Design and Verbal Fluency (0.039), Attention/Working Memory (*p* = 0.009), Learning (*p* = 0.014), Memory (*p* = 0.028), and Global Function (*p* = 0.012). Trends for similar HIV effects were found in the Executive function (*p* = 0.055) and Speed of Information Processing (*p* = 0.064) (Fig. 1). Although incident depression does not appear to affect neuropsychological functioning in HIV-infected men [34, 35], due to the group difference in CES-D, we also evaluated how CES-D score might influence the cognitive score and confirmed that except for Fluency, all other domains that showed significant HIV-effects remained significant (data not shown). With or without HIV infection, those who chronically used MJ did not perform worse in any of these domains. Although no significant HIV-by-MJ interaction was observed in any cognitive domain, HIV + MJ users performed slightly and non-significantly better than HIV+ subjects in several domains, including Design and Verbal Fluency, Executive Function, and Speed of Information Processing (Fig. 1).

### HIV infection and chronic marijuana use on DTI metrics

**Fig. 2:**
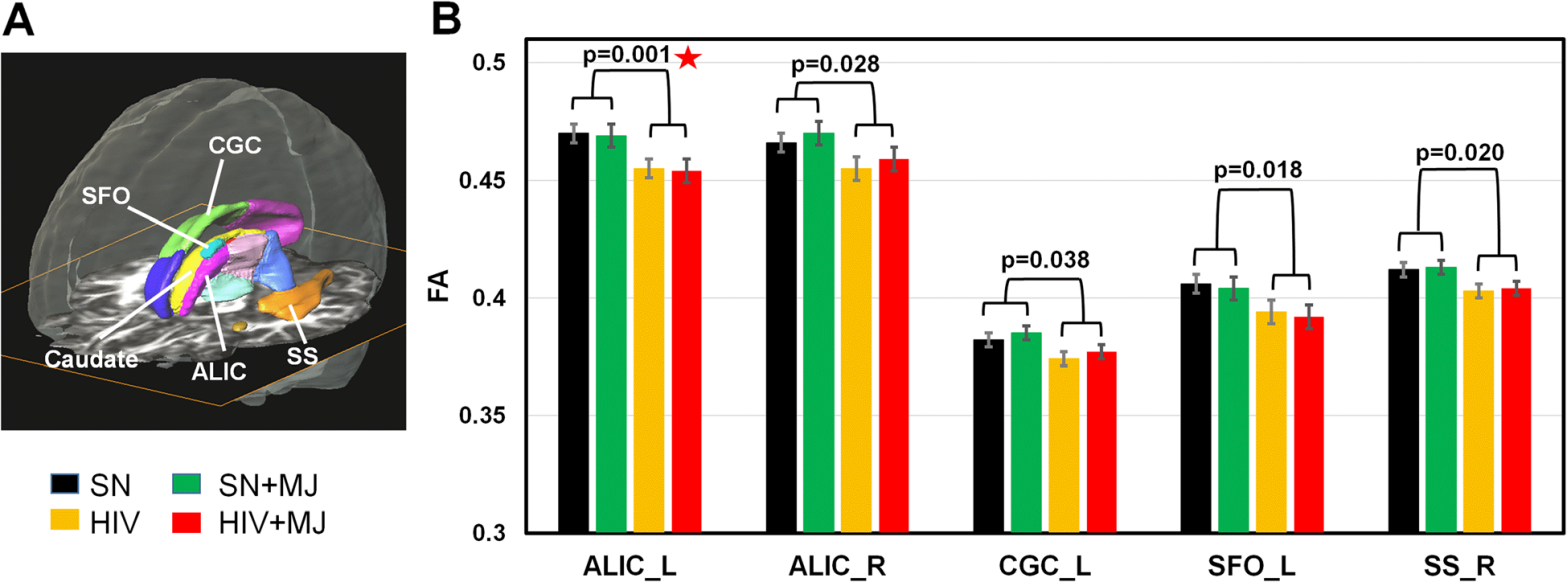
HIV+ participants showed lower regional fractional anisotropy (FA) than SN subjects on DTI. **A** Regions of interest automatically segmented for the FA measures that showed significant HIV effects are shown relative other structures. **B** Compared to SN participants, and regardless of MJ use status, HIV+ had lower FA in both right and left anterior limb of internal capsule (ALIC), left cingulate gyrus cingulum (CGC), left superior fronto-occipital fasciculus (SFO), and right sagittal stratum (SS). SN, HIV-seronegative nonuser group; SN + MJ, HIV-seronegative marijuana user group; HIV, HIV-seropositive nonuser group; HIV + MJ, HIV-seropositive marijuana user group. Red star: *p* value remained significant after Holm-Bonferroni correction for multiple comparisons

**Fig. 3:**
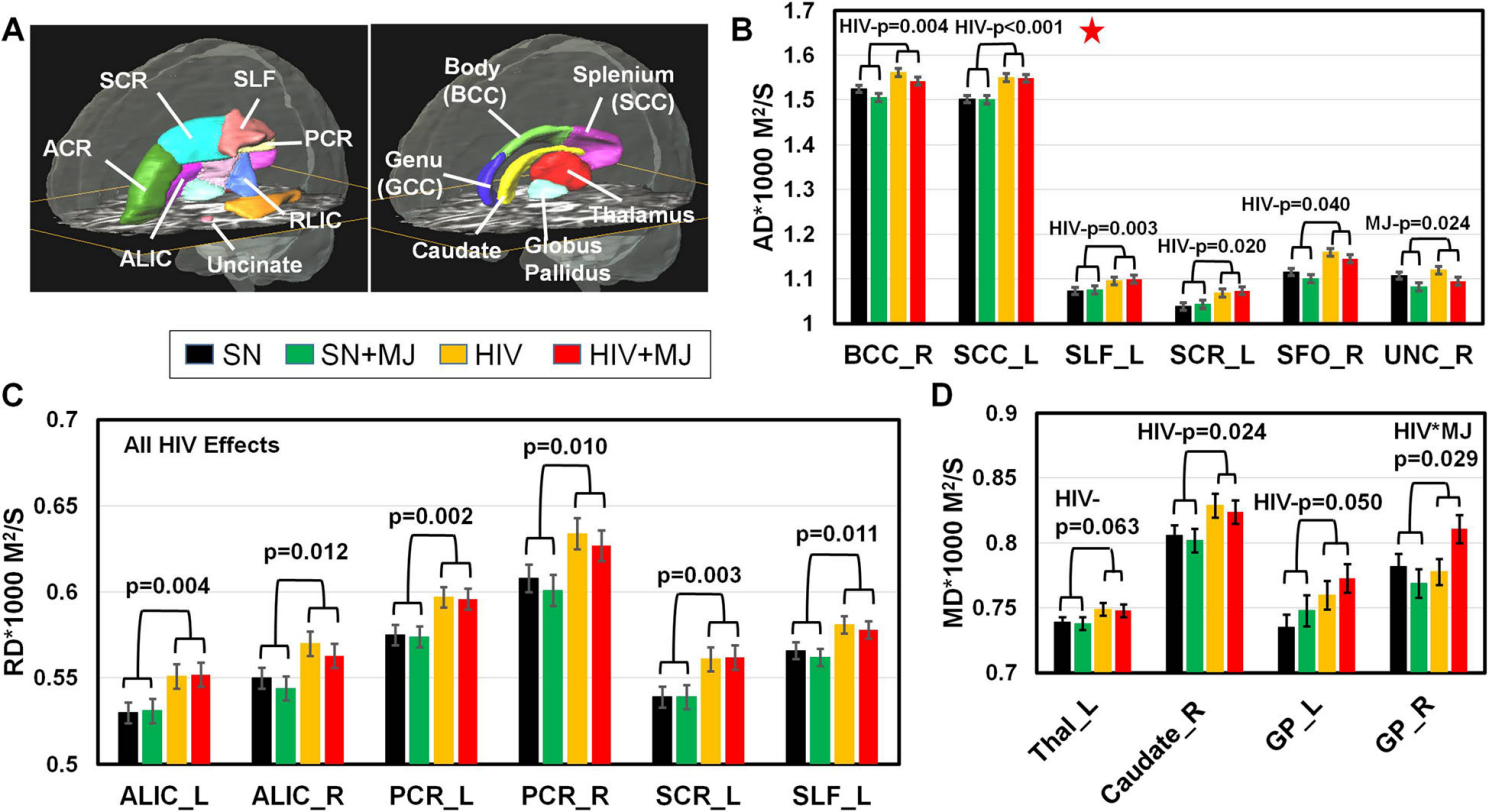
Group differences in DTI diffusivities. **A** Regions of interest automatically segmented for DTI measures that showed most of the significant HIV or MJ main effects. **B** Regardless of MJ use status, HIV+ group showed higher axial diffusivity (AD) in the right body of the corpus callosum (BCC), left splenium of the corpus callosum (SCC), left superior longitudinal fasciculus (SLF), left superior corona radiata (SCR), and right superior fronto-occipital fasciculus (SFO), than SN participants. However, regardless of HIV serostatus, MJ users had lower AD in the right uncinate fasciculus (UNC, *p* = 0.024) than nonusers. **C** Compared to SN controls and regardless of MJ use status, HIV+ had higher radial diffusivity (RD) than SN participants in the bilateral anterior limb of the internal capsule (ALIC), bilateral posterior corona radiata (PCR), left SCR, and left SLF, as well as 6 other brain regions (see Table 3). **D** Regardless of MJ use status, HIV+ group had higher mean diffusivity (MD) in the right caudate, left and right globus pallidus (GP), and left thalamus than SN groups. In additional, HIV-by-marijuana use showed an interaction effect in the right GP (*p* = 0.029); while HIV + MJ users had higher MD than HIV nonusers, SN-MJ users had lower MD than SN nonusers. SN, HIV-seronegative nonuser group; SN + MJ, HIV-seronegative marijuana user group; HIV, HIV-seropositive nonuser group; HIV + MJ, HIV-seropositive marijuana user group. Red star: *p* value remained significant after Holm-Bonferroni correction for multiple comparisons

Independent of MJ use, HIV+ participants had lower FA than SN controls in bilateral anterior lateral internal capsule (ALIC), the left cingulum (CGC_L), the left superior fronto-occipital fasciculus (SFO_L), and the right sagittal stratum (SS_R) (*p* values between 0.001 and 0.038); only ALIC_L remained significant after correction for multiple comparisons (Table 2; Fig. 2A, B). HIV+ group also had higher diffusivities (AD, RD, or MD) in multiple white matter and subcortical structures (Table 3, Fig. 3). For example, compared to SN subjects and regardless of MJ use, HIV+ had higher AD in the right BCC, left SCC, left SLF, left SCR, and right SFO (Fig. 3B), as well as higher RD in bilateral ALIC, bilateral PCR, left SCR, and left SLF (Fig. 3C), in addition to 6 other brain regions (Table 3). In the subcortical regions, HIV+ had higher MD in the right caudate, left and right GP, and a trend for higher MD in the thalamus (Table 3; Fig. 3D). The only brain region that showed an MJ use effect, regardless of HIV serostatus, was the right uncinate fasciculus, which showed lower AD in MJ users than nonusers (*p* = 0.024, Fig. 3B). In addition, an HIV-by-MJ interaction (*p* = 0.029) was observed for MD in the right GP; while SN + MJ had lower MD than SN nonusers, HIV + MJ had higher MD than HIV+ (Table 3, Fig. 3D). In addition to age, we also included percentage of lifetime tobacco users and percentage of regular alcohol use within the past month as covariates in the final model, since these two variables showed group differences, but all significant findings remained, and the interaction effect on the MD in right GP became more significant (Tables 2 and 3). Our exploratory analyses found no correlations between the DTI metrics that showed group differences and HIV-related clinical features (e.g., nadir CD4 and current CD4 counts and duration of HIV infection) or MJ usage patterns (age of onset, daily average use, duration and lifetime amount of MJ use).

**Table 2:** Fractional anisotropy in regions of interest (ROIs) that showed HIV effects

Brain region	Subject group				2-way ANCOVA main effect				
					Co-variate			HIV	MJ
	SN	SN + MJ	HIV	HIV + MJ	Age	% Recent regular alcohol use	% Lifetime tobacco smokers		
ALIC_L	0.47 ± 0.004	0.469 ± 0.004	0.455 ± 0.004	0.454 ± 0.004	0.014	0.472	0.965	0.001*	0.890
ALIC_R	0.466 ± 0.004	0.47 ± 0.005	0.455 ± 0.005	0.459 ± 0.005	0.031	0.296	0.708	**0.028**	0.508
CGC_L	0.382 ± 0.003	0.385 ± 0.004	0.374 ± 0.003	0.377 ± 0.003	0.009	0.243	0.610	**0.038**	0.493
CGH_L	0.368 ± 0.003	0.367 ± 0.003	0.363 ± 0.003	0.362 ± 0.003	0.077	0.434	0.987	0.099	0.630
PTR_R	0.455 ± 0.004	0.457 ± 0.005	0.447 ± 0.005	0.448 ± 0.005	0.002	0.357	0.076	0.094	0.797
SFO_L	0.406 ± 0.004	0.404 ± 0.005	0.394 ± 0.005	0.392 ± 0.005	0.041	0.360	0.680	**0.018**	0.749
SFO_R	0.418 ± 0.004	0.416 ± 0.005	0.408 ± 0.004	0.406 ± 0.005	0.005	0.237	0.817	0.054	0.657
SS_R	0.412 ± 0.003	0.413 ± 0.003	0.403 ± 0.003	0.404 ± 0.003	0.000	0.443	0.550	**0.020**	0.804
Caudate_L	0.357 ± 0.003	0.361 ± 0.003	0.35 ± 0.003	0.355 ± 0.003	0.066	0.659	0.007	0.065	0.215

All uncorrected *p* values < 0.05 from two-way ANCOVA are shown; the other 11 ROIs not listed showed no HIV or MJ effects. No HIV-by-MJ interaction was found for FA measurement in any of the ROIs. Bold: uncorrected *p* value < 0.05 for HIV or MJ main effects or their interaction

*ALIC* anterior limb of the internal capsule, *CGC* cingulum, cingulate gyrus part, *CGH* cingulum, hippocampal part, *PTR* posterior thalamic radiation, *PLIC* posterior limb of the internal capsule, *SS* sagittal stratum, *SFO* superior fronto-occipital fasciculus, *R* right, *L* left, *HIV* HIV seropositive, *SN* HIV seronegative, *MJ* marijuana

*p values remained significant after Holm correction for multiple comparisons

**Table 3:** Diffusivities in regions of interest (ROIs) that showed HIV or MJ effects

Brain region	Subject group				2-way ANCOVA					
					Co-variate			Main effect		HIV × MJ
	SN	SN + MJ	HIV	HIV + MJ	Age	ALC	TS	HIV	MJ	
Axial diffusivity										
BCC_R	1.524 ± 0.01	1.505 ± 0.012	1.561 ± 0.011	1.542 ± 0.011	0.001	0.418	0.608	**0.004**	0.148	ns
SCC_L	1.502 ± 0.011	1.5 ± 0.012	1.55 ± 0.012	1.548 ± 0.012	< 0.001	0.457	0.932	< 0.001*	0.899	ns
SLF_L	1.072 ± 0.006	1.075 ± 0.007	1.095 ± 0.007	1.099 ± 0.007	0.001	0.734	0.305	**0.003**	0.690	ns
UNC_R	1.107 ± 0.008	1.082 ± 0.009	1.119 ± 0.009	1.094 ± 0.009	0.129	0.951	0.434	0.241	**0.024**	ns
CGC_R	1.175 ± 0.01	1.17 ± 0.012	1.185 ± 0.011	1.18 ± 0.011	0.001	0.406	0.034	0.442	0.694	ns
EC_L	1.106 ± 0.008	1.098 ± 0.009	1.123 ± 0.009	1.115 ± 0.009	< 0.001	0.790	0.003	0.066	0.422	ns
SCR_L	1.038 ± 0.011	1.043 ± 0.012	1.068 ± 0.012	1.073 ± 0.012	< 0.001	0.697	0.056	**0.020**	0.727	ns
SFO_R	1.115 ± 0.018	1.1 ± 0.02	1.159 ± 0.019	1.145 ± 0.02	0.001	0.635	0.243	**0.040**	0.538	ns
Radial diffusivity										
ALIC_L	0.53 ± 0.006	0.531 ± 0.007	0.551 ± 0.007	0.552 ± 0.007	< 0.001	0.829	0.279	**0.004**	0.889	ns
ALIC_R	0.55 ± 0.006	0.544 ± 0.007	0.57 ± 0.007	0.563 ± 0.007	< 0.001	0.499	0.441	**0.012**	0.461	ns
PCR_L	0.575 ± 0.006	0.574 ± 0.006	0.597 ± 0.006	0.596 ± 0.006	< 0.001	0.841	0.026	**0.002**	0.943	ns
PCR_R	0.608 ± 0.008	0.601 ± 0.009	0.634 ± 0.009	0.627 ± 0.009	< 0.001	0.271	0.070	**0.010**	0.488	ns
SCR_L	0.539 ± 0.006	0.539 ± 0.007	0.561 ± 0.007	0.562 ± 0.007	0.003	0.685	0.057	**0.003**	0.971	ns
SCR_R	0.533 ± 0.007	0.533 ± 0.008	0.55 ± 0.007	0.551 ± 0.007	0.002	0.917	0.057	**0.030**	0.966	ns
SFO_L	0.554 ± 0.01	0.562 ± 0.012	0.581 ± 0.011	0.59 ± 0.011	0.001	0.935	0.235	**0.028**	0.509	ns
SFO_R	0.556 ± 0.011	0.553 ± 0.013	0.59 ± 0.012	0.587 ± 0.012	< 0.001	0.492	0.336	**0.013**	0.807	ns
PLIC_L	0.453 ± 0.005	0.458 ± 0.005	0.463 ± 0.005	0.467 ± 0.005	0.010	0.755	0.446	0.093	0.464	ns
RLIC_L	0.57 ± 0.004	0.57 ± 0.005	0.582 ± 0.005	0.582 ± 0.005	0.005	0.761	0.513	**0.022**	0.989	ns
EC_L	0.602 ± 0.005	0.593 ± 0.006	0.618 ± 0.006	0.609 ± 0.006	< 0.001	0.396	0.013	**0.019**	0.213	ns
IFO_L	0.593 ± 0.006	0.587 ± 0.006	0.606 ± 0.006	0.6 ± 0.006	0.001	0.600	0.107	0.057	0.367	ns
SLF_L	0.566 ± 0.005	0.562 ± 0.006	0.581 ± 0.005	0.578 ± 0.006	0.016	0.366	0.064	**0.011**	0.578	ns
SLF_R	0.579 ± 0.007	0.571 ± 0.008	0.598 ± 0.007	0.591 ± 0.007	0.008	0.366	0.044	**0.020**	0.412	ns
Mean diffusivity										
Thal_L	0.739 ± 0.004	0.738 ± 0.005	0.749 ± 0.005	0.748 ± 0.005	0.002	0.166	0.414	0.063	0.934	ns
Caudate_R	0.806 ± 0.008	0.802 ± 0.009	0.829 ± 0.009	0.824 ± 0.009	0.019	0.788	0.102	**0.024**	0.693	ns
GP_L	0.735 ± 0.01	0.748 ± 0.012	0.76 ± 0.011	0.773 ± 0.011	0.647	0.027	0.945	**0.050**	0.328	ns
GP_R	0.782 ± 0.01	0.769 ± 0.011	0.778 ± 0.01	0.811 ± 0.011	0.129	0.458	0.213	0.772	0.412	**0.029**

All uncorrected *p* values < 0.05 from two-way ANCOVA are shown. *p* values < 0.05 for HIV or MH main effects of their interaction are shown in bold

*ALIC* anterior limb of the internal capsule, *BCC* body of the corpus callosum, *CGC* cingulum, cingulate gyrus part, *EC* external capsule, *GP* globus pallidus, *IFO* inferior fronto-occipital fasciculus, *PCR* posterior corona radiata, *PLIC* posterior limb of the internal capsule, *RLIC* retrolenticular part of the internal capsule, *SCC* splenium of the corpus callosum, *SCR* superior corona radiata, *SLF* superior longitudinal fasciculus, *SFO* superior fronto-occipital fasciculus, *Thal* thalamus, *UNC* uncinate fasciculus, *ns* not significant (*p* ≥ 0.05)

*p values remained significant after Holm correction for multiple comparisons

### Abnormal DTI metrics predicted abnormal cognitive domain *Z*-scores

**Fig. 4:**
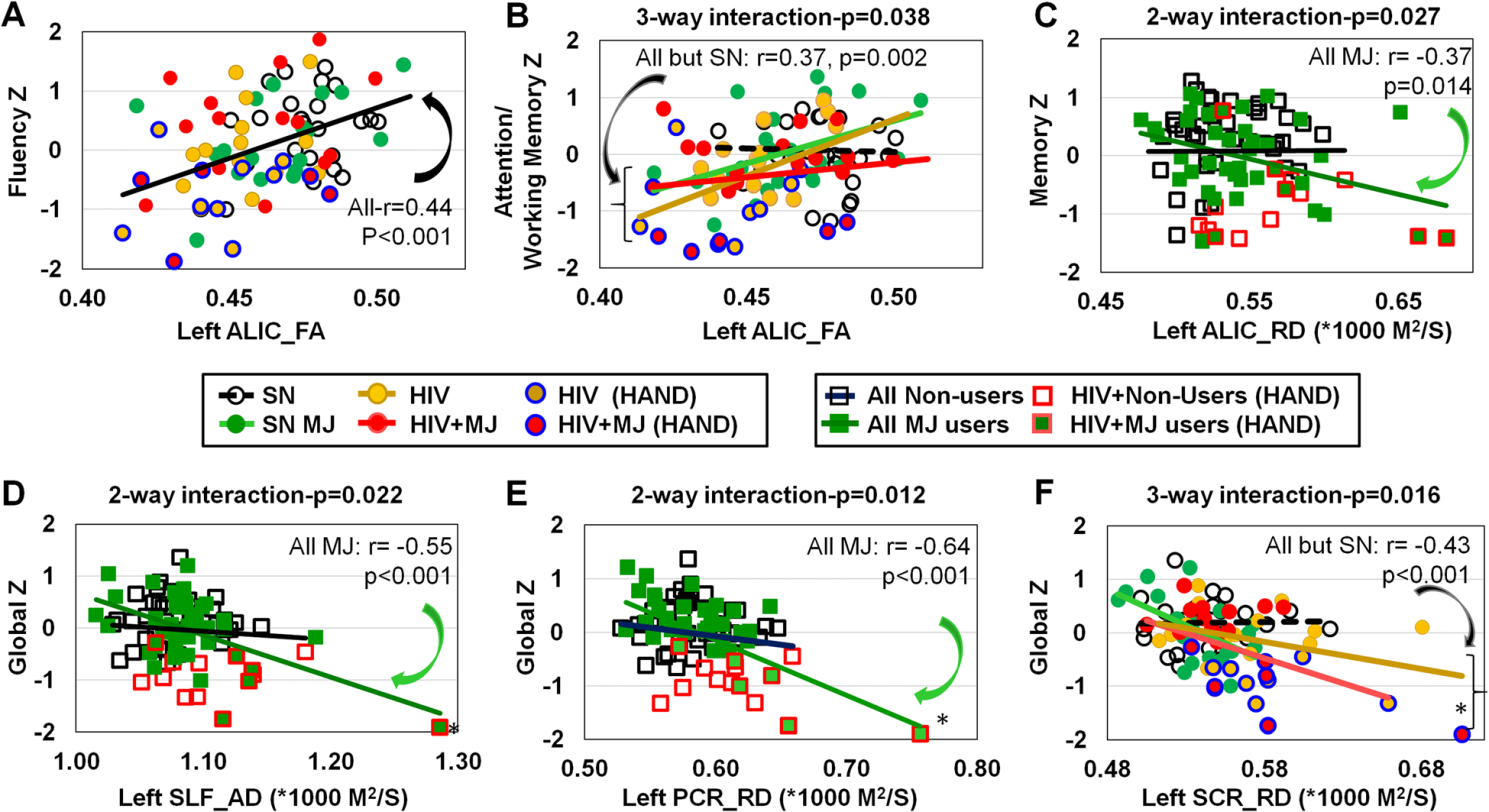
DTI, metrics predicted cognitive performance on domain *Z*-scores across subject groups. HIV+ participants with HAND are further indicated with dark blue borders on the circles or red borders on the squares. **A**, **B** Lower FA in the left anterior limb of the internal capsule (ALIC) predicted poorer Fluency across all participants and poorer Attention and Working Memory in all participants except for the SN nonusers (interaction *p* = 0.038). **C** Higher radial diffusivity (RD) in the left ALIC also predicted poorer Memory in MJ users regardless of HIV serostatus but not in non-MJ users (interaction *p* = 0.027). **D**, **E** Lower Global *Z*-scores were predicted by higher AD in left superior longitudinal fasciculus (SLF) and higher RD in left posterior corona radiata (PCR). The asterisk (*) indicates both interaction *p* values remained significant when the highest diffusion measures were removed (left SLF_AD: *p* = 0.040; left PCR_RD: *p* = 0.016). **F** Lower Global *Z*-score was also predicted by higher RD in the left superior corona radiata (SCR) in all participants except for the SN nonuser group (interaction *p* = 0.016). The asterisk (*) indicates the 3-way interaction *p* value for the SCR_L became even more significant if the highest diffusion point is removed (*p* = 0.002). SN, HIV-seronegative nonuser group; SN + MJ, HIV-seronegative marijuana user group; HIV, HIV-seropositive nonuser group; HIV + MJ, HIV-seropositive marijuana user group. HAND (HIV-associated neurocognitive disorder)

We evaluated whether DTI metrics that showed group or interactive effects also predicted the cognitive performance. Lower left ALIC_FA predicted lower Fluency across all participants (*r* = 0.44; *p* < 0.001, Fig. 4A) and lower Attention/Working Memory in all except SN subjects (*p* = 0.037; *p* = 0.002, Fig. 4B). Higher left ALIC_RD also predicted lower Memory *Z*-scores in all MJ users (*r* = − 0.037, *p* = 0.014) but not in nonusers (interaction *p* = 0.027, Fig. 4C). Furthermore, lower Global *Z*-scores in all MJ users, but not in nonusers, were predicted by higher left SLF_AD (*r* = − 0.55, *p* < 0.001; interaction *p* = 0.022, Fig. 4D) and higher left PCR_RD (*r* = − 0.64, *p* < 0.001; interaction *p* = 0.012, Fig. 4E). Lastly, Global *Z*-scores across all participants, except for SN subjects, were predicted by higher left SCR_RD (3-way interaction *p* = 0.016; Fig. 4F)

### Age-related changes in DTI

**Fig. 5:**
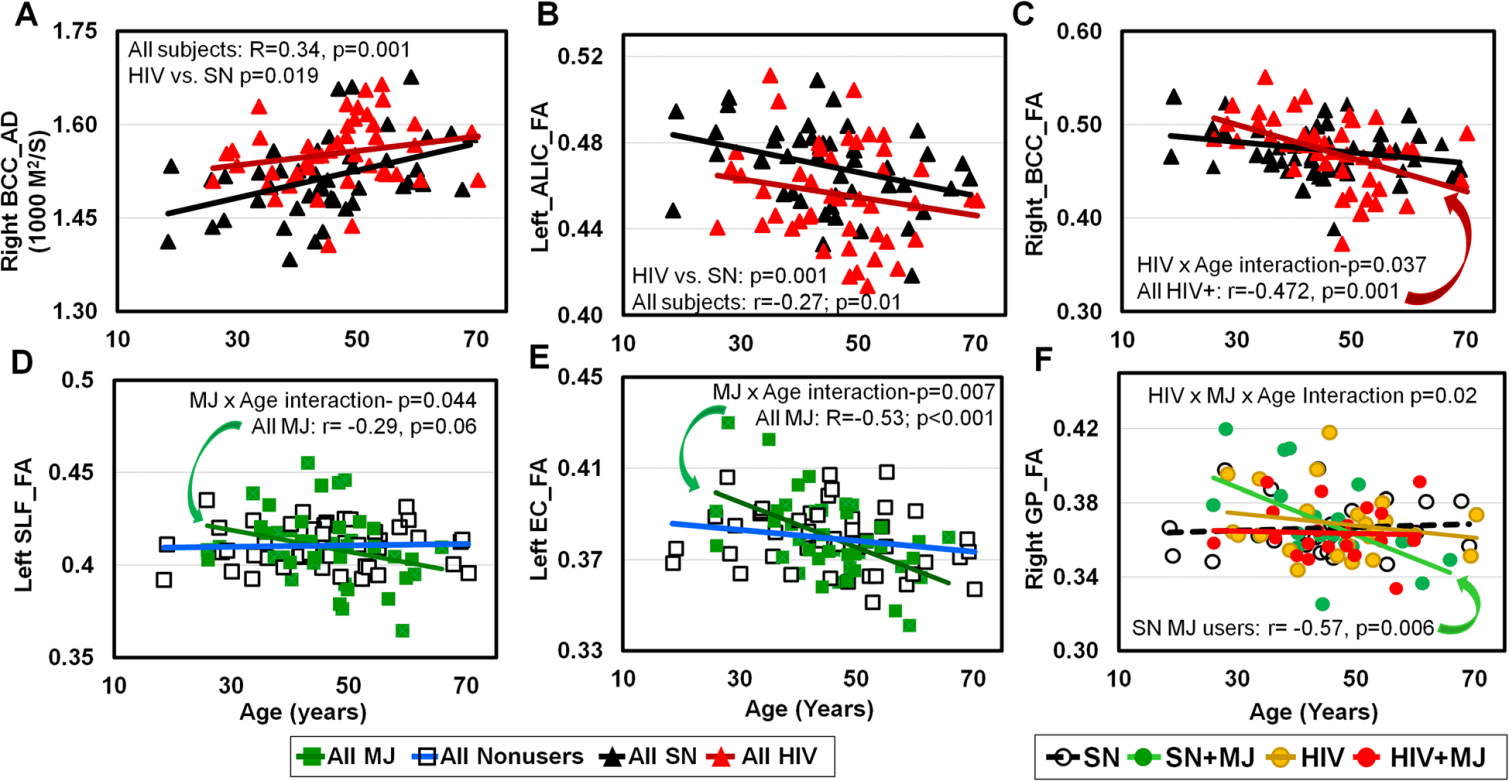
Correlations between DTI metrics and age. **A** Despite the higher AD in HIV than SN (regardless of MJ use), the two groups showed similar slopes for age-dependent increases in AD. The AD in the body of the corpus callosum (BCC) is illustrated as an example. **B** Although HIV+ had lower FA than SN, both groups (regardless of MJ use) showed similar age-dependent declines; see in left anterior limb of the internal capsule (ALIC) FA for example. **C** Greater age-related declines in the BCC FA were observed in HIV+ individuals, with or without MJ use, than SN subjects (HIV × age-interaction *p* = 0.037). **D**, **E** Compared to all nonusers (blue line), MJ users with or without HIV (dark green line) showed greater age-related declines in FA in left superior longitudinal fasciculus (SLF: MJ × age-interaction *p* = 0.044) and left external capsule (EC: MJ × age-interaction *p* = 0.007). **F** Age-related decline in the right GP_FA was observed in SN-MJ users only (3-way interaction *p* = 0.02); the other three groups already showed lower levels of FA at younger ages. SN, HIV-seronegative nonuser group; SN + MJ, HIV-seronegative marijuana user group; HIV, HIV-seropositive nonuser group; HIV + MJ, HIV-seropositive marijuana user group

Although HIV+ showed lower FA and higher diffusivities than SN in multiple white matter and subcortical regions, regardless of MJ use, HIV+ and SN showed similar age-dependent decreases in FA in 5 brain regions (left and right ACR, left and right GCC, and right SS) and age-dependent increases in diffusivities in 16/44 regions (including left and right hemispheres, *p* values between 0.001 and 0.049, data not shown, except for BCC_AD and ALIC_FA, Fig. 5A, B). In addition, independent of MJ use, HIV+ showed greater age-related decline in the right BCC_FA than SN (HIV × age-interaction *p* = 0.037; Fig. 5C). Furthermore, regardless of HIV serostatus, MJ users showed greater age-related decline than nonusers in left SLF_FA (MJ × age-interaction *p* = 0.044; Fig. 5D) and in left EC_FA (MJ × age-interaction *p* = 0.007; Fig. 5E). Lastly, age-related decline in the right GP_FA was found only in MJ users (HIV × MJ × age-interaction *p* = 0.02, Fig. 5F).

## Discussion

The main findings of this study are as follows: (1) As hypothesized, compared to SN and regardless of MJ use status, the HIV+ group had poorer cognitive performance despite cART in all participants. (2) In contrast, regardless of HIV serostatus, MJ users and nonusers had similar cognitive performance; hence, no interactions or additive deleterious effects were found in HIV + MJ on cognitive test scores. (3) DTI measures in HIV+ group, with or without MJ use, had lower FA and higher diffusivities than SN controls in multiple white matter and subcortical brain regions, indicating greater neurodegeneration and neuroinflammation. (4) However, regardless of HIV serostatus, MJ users had lower AD, suggesting lesser fiber integrity, only in the right UNC than nonusers. Furthermore, we observed an HIV-by-MJ interaction in the right GP_MD, indicating differential MJ effects on neuroinflammation in this brain region of HIV patients compared to SN controls.

### Cognitive performance in chronic MJ users with and without HIV infection

The poorer performance in HIV+ compared to SN controls, regardless of MJ use, in the domains of Design and Verbal Fluency, Attention/Working Memory, Learning, Memory, and Global Functioning is consistent with prior studies in HIV+ individuals [1]. These persistent cognitive abnormalities despite cART were attributed primarily to ongoing neuroinflammation [23–26]. Also similar to prior reports [7–10], regardless of HIV status, MJ users had similar performance across all cognitive domains as nonusers. The lack of cognitive deficits in our adult MJ users suggests little or no neurotoxic effects associated with chronic MJ use, which is supported by the lack of decline in IQ in adult onset MJ users [14]. In contrast, the developing brain of adolescents may be more vulnerable to the neurotoxic effects of MJ. Earlier onset or regular (weekly) MJ use was associated with lower cognition [15, 16] and decline in IQ and cognitive function (between ages 13 and 38 years) [14], while earlier age of first recreational MJ use was associated with lower FA and higher RD in the SLF, ILF, and forceps major and minor [27]. Furthermore, greater gray matter volumes in bilateral posterior cingulate, lingual gyri, and cerebellum were found in 14 years old adolescents who had only one or two instances of cannabis use compared to matched cannabis-naïve controls [27].

In the current study, although no significant HIV-by-MJ interaction was found in any of the cognitive domains, SN + MJ tended to have poorer performance than SN nonusers in Learning, Memory, and motor domains, while HIV + MJ tended to perform better than HIV+ nonusers in Design and Verbal Fluency, Executive Function, and Speed of Information Processing. These trends are consistent with a recent large study that found MJ use was associated with lower odds of neurocognitive impairment and higher verbal fluency and learning performance, in PLWH, but not in the SN participants [11]. This paradoxical effect of MJ use in SN and HIV+ individuals might be related to the anti-inflammatory effects from some of the MJ constituents on the neuroinflammation in PLWH [36, 37]. For example, Δ9-THC suppresses cytokine-induced T-cell activation [36, 37] and lowers the monocyte-derived proinflammatory factor IP-10 in vitro [37]. Furthermore, MJ using HIV+ participants showed faster decline of cellular HIV DNA levels during the first 4 months of cART, compared with those who did not use MJ or used other substances [38]. In addition, HIV+ light MJ users had better verbal fluency than SN light users [9], but this advantage was not found in HIV+ heavy MJ users [8], how the dosage and the potency of Δ9-THC in MJ, which has quadrupled in the past two decades [39], may impact cognition in PLWH will need to be evaluated in future studies.

### DTI metrics and neuroinflammation in chronic MJ users with and without HIV infection

Consistent with prior DTI studies [2, 40–42], our HIV+ participants, regardless of MJ use, had lower FA and/or higher diffusivities in the corpus callosum, coronal radiata, internal capsule, the cingulum, SLF, SFO, and other white matter tracts. The lower FA and higher diffusivities in HIV+ individuals most likely reflect disrupted white matter microstructure, perhaps due to neurodegeneration and chronic neuroinflammation induced by ongoing HIV+ infection. Our HIV+ participants also showed higher MD in subcortical gray matter (caudate, globus pallidus, and thalamus), which suggests possible demyelination in these subcortical regions. The elevated caudate_MD was also reported in patients with early HIV infection [43], while the elevated GP_MD in our HIV subjects would be consistent with the 18 kDa translocator protein (TSPO) binding, indicating microglial activation, in this brain region of virally suppressed HIV patients [44]. Furthermore, relatively lower FA in the globus pallidus, along with poorer motor skills, was also found in HIV+ women but not HIV+ men [45].

In contrast, the right uncinate fasciculus (UNC) was the only brain region that showed abnormally lower AD in MJ users, regardless of HIV status. The lower AD in the UNC indicates reduced water movement along the axonal fibers, which might results from lesser axonal fiber density, accumulated cellular debris from damaged axonal, or extracellular space tortuosity [46]. In preclinical studies, reduced AD was consistently found at early stages of brain injury from models of multiple sclerosis and correlated with the axonal damage or loss [46, 47]. Furthermore, the UNC in MJ users was found to have reduced FA and elevated MD [25], as well as shorter than normal fiber bundle [48]. Lower FA in the UNC was also associated with higher apathy scores in MJ users [25]. The UNC fasciculi are long-range projecting fibers that connect the orbitofrontal cortex with entorhinal and fusiform cortices, which have densely localized CB1 receptors that are target receptors for Δ9-THC [48]. Lower AD in the UNC might indicate lesser connectivity among these regions, which were found to be abnormally thinner and associated with poorer verbal memory in cannabis users [48].

The HIV-by-MJ interaction effect found in the right globus pallidus, with relatively lower MD in SN + MJ users but relatively higher MD in the HIV + MJ users, parallel the interactive effects observed in a proton MR spectroscopy (^1^H-MRS) study, with relatively lower levels of myoinositol, a glial marker, in the basal ganglia of SN + MJ users but relatively higher myoinositol levels in HIV + MJ users [7]. In HIV+ patients, higher diffusivity was associated with higher myoinositol level, indicating greater neuroinflammation, which in turn correlated with poorer cognitive performance [49]. Therefore, this interactive effect suggests that while chronic MJ use suppressed glial activation in the GP of SN subjects, chronic MJ use promoted glial activation in HIV+ users. Although DTI or ^1^H-MRS cannot determine the glial cell types or cellular processes involved with such glial activation, multiple in vitro or in vivo rodent or macaque HIV models demonstrated modulations of the microglial response by endocannabinoids as well as exogenous cannabinoids, such as Δ9-THC, see review [6]. For instance, in most rodent HIV models, endocannabinoid CB1/CB2 or CB2 receptor agonists [50, 51] or inhibitors of the degradation enzymes for endocannabinoids [52] led to decreased gp-120 induced inflammatory interleukin-1β and/or activation and upregulation of CB2 receptors, which are located on microglia, and ultimately suppressed the inflammatory processes associated with microglial activation and attenuated or mitigated HIV-associated neurotoxicity [6]. Another study that used a GFAP/GP120/FAAH-/- mouse model also demonstrated decreased astrogliosis with improved neurogenesis due to the decreased endocannabinoid degradation by fatty acide amide hydrolase (FAAH) [53]. The only study that administered Δ9-THC to HIV-infected SCID mice found higher viral load, upregulated CCR5 expression, and greater HIV+ cells with longer THC exposure [54]. Furthermore, in simian immunodeficiency virus (SIV)-infected macaques, Δ9-THC administered prior to SIV infection produced dose-dependent cognitive slowing, while chronic Δ9-THC after the SIV infection produced tolerance to the behavioral effects [55]. Another SIV model found that Δ9-THC before and after SIV infection slowed disease progression and decreased inflammation, as well as increased BDNF and decreased proinflammatory cytokines in the striatum [56]. Hence, although the majority of the cell culture and rodent models of HIV found endocannabinoids to have neuroprotective and anti-inflammatory effects, studies that used Δ9-THC in rodents and non-human primates were less clear. Future studies with more specific glial markers to assess these possible differential effects of MJ use on neuroinflammation in the GP between PLWH and SN are needed.

### DTI metrics predicted cognitive performance in HIV+ individuals and MJ users

Microstructural abnormalities predicted poorer performance on some cognitive function in our participants. Specifically, lower FA in ALIC, suggesting lesser fiber coherence in this tract, predicted lower performance on Design and Verbal Fluency across all participants, and lower performance on Attention/Working Memory among our HIV+ and MJ user subjects. In addition, we found higher diffusivities in several white matter tracts (ALIC, SLF, PCR, SCR) in our MJ users that predicted poorer memory and Global *Z*-scores, which is similar to the findings that higher corona radiata diffusivity was associated with slower processing speed in the aging population [57] or with poorer learning in HIV+ participants [41]. Lower fiber coherence and higher diffusivities were often reported in brain disorders with neuroinflammation and correlated with microglial activation and cognitive deficits. For instance, microglial activation, as shown by greater TSPO tracer [(11)C]PBR28 binding correlated with higher diffusion on DTI and greater cognitive deficits in HIV patients [44], while greater ionized calcium-binding adaptor (iba-1) and lower synaptophysin staining in brain tissues also correlated with greater diffusion on DTI and cognitive impairments in a mouse model of HIV [58]. Even in HIV patients who were virally suppressed, [11C]DPA-713-TSPO binding also predicted poorer cognitive performance in multiple cognitive domains [59]. Lastly, 18F-DPA714-TSPO-binding in SIVsm804E-infected rhesus macaques also correlated with microglial activation assessed from iba1 staining in brain tissue, along with alterations in CSF viral load, CSF levels of monocyte chemoattractant protein 1 (MCP-1), tumor necrosis factor alpha (TNF-α), and various inflammatory cytokines [60]. Taken together, these human and preclinical imaging studies demonstrated strong relationships between microglial activation, ongoing neuroinflammation, and cognitive dysfunction. Microglial activation, increased proinflammatory cytokine production, and a reduction in synaptic density are key pathological features associated with HIV-associated neurocognitive disorders (HAND).

Although the exact mechanisms for how microglial activation may lead to cognitive disorders remain unknown, one mechanism involves excitotoxic neuronal injury from increased extracellular glutamate concentration. The increased glutamate may result from upregulation of glutamate-generating enzyme glutaminase [61], which are found HIV-infected microglia and macrophages, and are potentiated by interferons from the innate immune responses [62], as shown in postmortem brain tissues of patients with HIV dementia [61, 63]. Furthermore, the proinflammatory cytokine tumor necrosis factor inhibits the reuptake of glutamate by activated astrocytes [64], leading to incomplete recycling of glutamate back to glutamine. This incomplete recycling would led to reduced intraneuronal glutamate levels, as observed on ^1^H-MRS studies, especially in HIV patients with cognitive deficits [65]. These imaging studies, along with preclinical and postmortem studies in HIV patients, documented that microglial and astroglial activation are both involved with the neuroinflammatory cascades that are amplified by toxic HIV-viral proteins, contributing to glutamate-mediated excitotoxic neuronal injury and cognitive dysfunction [66].

The relationships between marijuana use, microglial activation, and cognitive dysfunction are less clear. Two cannabinoids, Δ9-THC and cannabidiol (CBD), are present in marijuana, and both may decrease the production and release of proinflammatory cytokines, including interleukin-1beta (IL-1β), interleukin-6, and interferon (IFN)beta, from LPS-activated microglial cells [67], which would support the anti-inflammatory effects of marijuana. However, in mice, subchronic administration of THC activated cerebellar microglia and increased the expression of neuroinflammatory markers, including IL-1β, which in turn correlated with deficits in cerebellar conditioned learning and fine motor coordination [68]. Collectively, all of these studies indicate that greater diffusivity observed on DTI likely reflect ongoing microglial activation and neuroinflammation, which may ultimately lead to poorer cognitive performance.

### Age-related and MJ-related changes in DTI metrics in HIV+ individuals and MJ users

Our HIV+ participants had greater than normal age-dependent declines in FA, suggesting accelerated age-related loss of fiber integrity, in the corpus callosum regardless of MJ usage; this finding is consistent with those in prior DTI studies [40, 69]. In addition, we observed greater than normal age-related FA decline in the SLF and EC of MJ users regardless of HIV serostatus, which is also consistent with the greater than normal age-related FA decline in multiple white matter regions in MJ users [24]. Lastly, age-dependent decline was also observed in the right globus pallidus FA only in SN-MJ users, which indicates lesser microstructural integrity in these older MJ users. However, due to the limited sample size in each of the subgroups, these exploratory observations will need to be confirmed in future studies.

### Limitations

Our study has several limitations. (1) Our cohort included primarily men; therefore, we were not able to assess sex-specific differences on brain microstructure in relation to the possible additive or interactive effects of HIV infection and chronic MJ use. (2) Since this is a cross-sectional study, we could not determine the causality of chronic MJ use on altered DTI metrics or cognitive deficits in HIV+ individuals. Future longitudinal studies are necessary to further delineate the independent and combined effects of chronic MJ use and HIV infection on brain microstructure. (3) Self-report of MJ use or other substances used may be inaccurate or under-reported and might have confounded our results. (4) Despite cART in all HIV patients, a few patients still had detectable viral loads, which may be due to drug resistant mutations of the virus in these individuals. Therefore, the cognitive and DTI results may vary in those with persistent viral replication, which should be evaluated in future larger studies. (5) This higher than expected number of SN with HAND-equivalent cognitive status (21–27%) may be due to a selection bias, since individuals with subject cognitive complaints might have sought out research studies that provided free brain MRI scans and cognitive assessments. Having more HAND-equivalent SN control subjects might have minimized the cognitive group differences with the other subject groups; however, our SN and SN + MJ groups showed relatively normal *Z*-scores across the domains, except for slightly slower motor performance in the SN + MJ group.

## Conclusions

Adding to previous studies that did not find additional adverse effects of chronic MJ use on cognition [7–9], brain morphometry [8], and clinical outcomes, such as viral load, CD4 cell count, and total mortality [19, 21] in HIV+ participants, our findings suggest that chronic MJ use has no additional negative influence on neurocognitive deficits in PLWH. However, the lower AD in the UNC of MJ users suggests axonal loss in this white matter tract that connects to CB1 receptor rich brain regions that are involved in verbal memory [48] and emotion. Furthermore, the interactive effect on MD in the globus pallidus suggests that MJ use may have an anti-inflammatory effect in SN subjects but might exacerbate the neuroinflammation in this brain region in HIV patients. Furthermore, the greater than normal age-dependent FA declines in several white matter tracts, and in the GP in SN-MJ users, suggests that older MJ users may eventually have lesser neuronal integrity in these brain regions.

## Data Availability

The datasets used and/or analyzed during the current study are available from the corresponding author on reasonable request.

## Abbreviations

ACR: Anterior corona radiata
AD: Axial diffusivity
ALIC: Anterior limb of the internal capsule
AN(C)OVA: Analysis of (co-) variance
BCC: Body of the corpus callosum
cART: Combination antiretroviral therapy
CC: Corpus callosum
CES-D: Center for Epidemiological Studies–Depression Scale
CGC: Cingulum, cingulate gyrus part
CGH: Cingulum, hippocampal part
D-KEFS: Delis-Kaplan Executive Function System
DTI: Diffusion tensor imaging
EC: External capsule
FA: Fractional anisotropy
GCC: Genu of the corpus callosum
GP: Globus pallidus
HIV+: HIV seropositive
HIV + MJ: HIV-seropositive marijuana user group
Iba1: Ionized calcium-binding adaptor molecule
IFO: Inferior fronto-occipital fasciculus
ILF: Inferior longitudinal fasciculus
IQ: Intelligence quotient
ISP: Index of Social Position
MD: Mean diffusivity
MJ: Marijuana
PCR: Posterior corona radiata
PLIC: Posterior limb of the internal capsule
PLWH: People living with HIV
PTR: Posterior thalamic radiation
RAVLT: Rey Auditory Verbal Learning Test
ROCFT: Rey-Osterreith Complex Figure Test
RD: Radial diffusivity
RLIC: Retrolenticular part of the internal capsule
SCC: Splenium of the corpus callosum
SCR: Superior corona radiata
SFO: Superior fronto-occipital fasciculus
SLF: Superior longitudinal fasciculus
SN: Seronegative (for HIV)
SN + MJ: Seronegative (for HIV) and marijuana user group
SS: Sagittal stratum
SUD: Substance use disorder
Thal: Thalamus
TSPO: Translocator protein
UNC: Uncinate fasciculus
WTAR: Wechsler Test of Adult Reading
